# New, Low–Molecular Weight Chemical Compounds Inhibiting Biological Activity of Interleukin 15

**DOI:** 10.3390/molecules28052287

**Published:** 2023-03-01

**Authors:** Piotr Krzeczyński, Małgorzata Dutkiewicz, Oliwia Zegrocka-Stendel, Bartosz Trzaskowski, Katarzyna Koziak

**Affiliations:** 1 Chemistry Section, Pharmacy, Cosmetic Chemistry and Biotechnology Research Group, Łukasiewicz Research Network–Industrial Chemistry Institute, Rydygiera 8, 01-793 Warsaw, Poland; 2 Department of Biochemistry and Nutrition, Centre for Preclinical Research and Technologies, Medical University of Warsaw, S. Banacha1b, 02-097 Warsaw, Poland; 3 Centre of New Technologies, University of Warsaw, S. Banacha 2c, 02-097 Warsaw, Poland

**Keywords:** IL–15, IL–15Rα, small–molecule IL–15Rα inhibitor, benzoic acid

## Abstract

Chronic overproduction of IL–15 contributes to the pathogenesis of numerous inflammatory and autoimmune disorders. Experimental methods used to reduce the cytokine activity show promise as potential therapeutic approaches to modify IL–15 signaling and alleviate the development and progression of IL–15–related diseases. We previously demonstrated that an efficient reduction of IL–15 activity can be obtained by selective blocking of the specific, high affinity subunit alpha of the IL–15 receptor (IL–15Rα) with small–molecule inhibitors. In this study, we determined the structure–activity relationship of currently known IL–15Rα inhibitors in order to define the critical structural features required for their activity. To validate our predictions, we designed, analyzed in silico, and assessed in vitro function of 16 new potential IL–15Rα inhibitors. All newly synthesized molecules were benzoic acid derivatives with favorable ADME properties and they efficiently reduced IL–15 dependent peripheral blood mononuclear cells (PBMCs) proliferation, as well as TNF–α and IL–17 secretion. The rational design of IL–15 inhibitors may propel the identification of potential lead molecules for the development of safe and effective therapeutic agents.

## 1. Introduction

Interleukin 15 (IL–15) is a member of the common receptor gamma chain (γ_c_) family, which also includes IL–2, IL–4, IL–7, IL–9, and IL–21. IL–15 is widely expressed and acts pleiotropically on many immune and non–immune cell types affecting their development and function [1]. To deliver its signal, IL–15 uses a heterotrimeric receptor composed of a specific, high affinity subunit alpha of the IL–15 receptor (IL–15Rα), IL–2/IL–15 specific receptor beta (IL–2/IL–15Rβ, CD122) and a common cytokine receptor gamma (γ_c_, CD123). Depending on the arrangement of the receptor units, the IL–15 signal can be delivered either in *cis* or *trans* mode. In *cis*–presentation, IL–15Rα and IL–2Rβ/γ_c_ are expressed on the surface of the same cell while in *trans*–mode, which dominates in vivo, IL–15Rα presents IL–15 to neighboring cells bearing IL–15Rβ/γ_c_.

Errors leading to IL–15 overproduction directly contribute to the pathogenesis of numerous inflammatory and autoimmune disorders, e.g., rheumatoid arthritis, psoriasis, multiple sclerosis, celiac disease, type 1 diabetes, and alopecia areata. Increased concentrations of IL–15 are also linked to T–cell leukemias and graft rejection. Targeting IL–15 has been shown to be a valuable therapeutic method in many preclinical studies and clinical trials, however, to date none of the potential IL–15 inhibitors have been approved for clinical use. The experimental approaches used to inhibit cytokine activity include soluble IL–15Rα [2,3,4], antibodies inhibiting IL–2/IL–15Rβ [5] or IL–15 [6,7], a peptide inhibitor of γ_c_ [8] or a modified IL–15 molecule with competitive antagonist activity [9]. We have previously demonstrated that selective blockage of IL–15Rα with small molecule inhibitors also leads to an efficient reduction of IL–15 activity. The efficacy of this approach correlates well with the significance of IL–15Rα in controlling IL–15 action. The accumulated evidence shows that IL–15 acts predominantly not as a monomer, but in complex with IL–15Rα. IL–15Rα is required as an intracellular chaperone for IL–15 secretion, complexed with IL–15 it prolongs the cytokine half–life, it is also necessary for optimal signaling through the IL–2Rβ/γ_c_ dimeric receptor [10].

In the current study, we attempted to define the critical structural features of IL–15Rα inhibitors required for their activity against IL–15Rα. We based our research on previously identified, first potential IL–15Rα inhibitors selected on the basis of in silico modelling, pharmacophores screening, and prediction of ADME properties [11]. Furthermore, to validate our estimates, we designed, synthesized, and subjected to in vitro analysis a group of 16 new compounds presented in this paper.

All tested molecules reduced IL–15 dependent responses with significantly higher efficacy compared to currently known IL–15Rα inhibitors. The data obtained show that the applied approach allows the design of small molecular inhibitors of IL–15 Rα and could provide the results necessary to progress toward the successful development of a therapeutic agent.

## 2. Results and Discussion

### 2.1. Structural Characterization of Active and Inactive IL–15 Rα Inhibitors from the Group of Benzoic Acid Derivatives

Among the previously described molecules identified through in silico modeling and pharmacophores screening as putative small molecule IL–15Rα inhibitors, a vast majority, i.e., 14 out of 16 molecules, are benzoic acid derivatives [11] (Table 1). However, only half of them, that is seven molecules, exhibit inhibitory activity against IL–15, as demonstrated in biological in vitro tests (Table 1A). In our attempts to identify structural features necessary for these compounds activity, we have revealed that most of the active molecules (**R11**–**R16**) were benzoic acids derivatives that contained either acyclic—succinate or maleate or cyclic—cyclohexyl or norbornane substituents bearing free carboxylic group. More specifically, **R11** and **R13** were aminomethylbenzoic acid derivatives, **R16** was a derivative of *p*–aminobenzoic acid, while R12 and R15 were derivatives of 3,5–diaminobenzoic acid.

**Figure 1:**
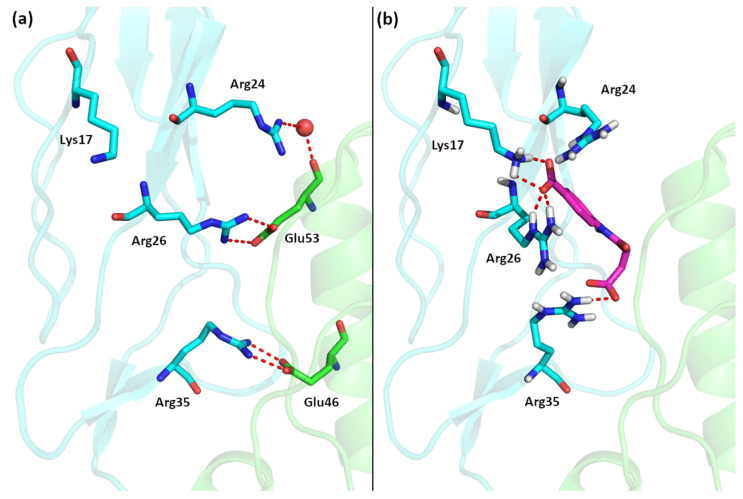
(**a**) The interface of the IL–15/IL–15Rα complex. (**b**) Best computational pose of **7a** docked to IL–15Rα (IL–15 in green, IL–15Rα in blue).

The very high prevalence of benzoic acid derivatives in the group of the IL–15Rα inhibitors is not surprising. The X–ray structure of the IL–15R/IL–15 complex reveals that the interaction between interleukin and its receptors is stabilized mainly by two salt bridges between two arginine moieties of IL–15Rα and two glutamic acid residues of IL–15 (Arg26–Glu53 and Arg35–Glu46), located approximately 8–9 Å from each other (Figure 1a) [12].

During our previous in silico search for potential IL–15Rα inhibitors we specifically targeted to disrupt these interactions; therefore, most of the identified compounds had either two chemical groups with a formal +1 charge with high affinity to IL–15 or two moieties bearing a formal –1 charge with the high affinity to IL–15Rα [11]. The high prevalence of compounds with these moieties is likely due to the fact that carboxylic groups are the most common negatively charged moieties in all organic systems and the presence of benzoic acid produces relatively rigid molecules able to target both arginine residues of IL–15Rα which are relatively far apart. It is also worth noting that in the structure of the entire IL–15/IL–15R quaternary complex the targeted region of IL–15Rα has the most basic character, as there are in total four basic residues (Lys17, Arg24, Arg26 and Arg35), therefore binding of the newly designed inhibitors to other parts of the IL–15/IL–15R complex, such as the β or γ subunits, is unlikely [13].

### 2.2. Design and In Silico Analysis of New Potential IL–15Rα Inhibitors

**Figure 2:**
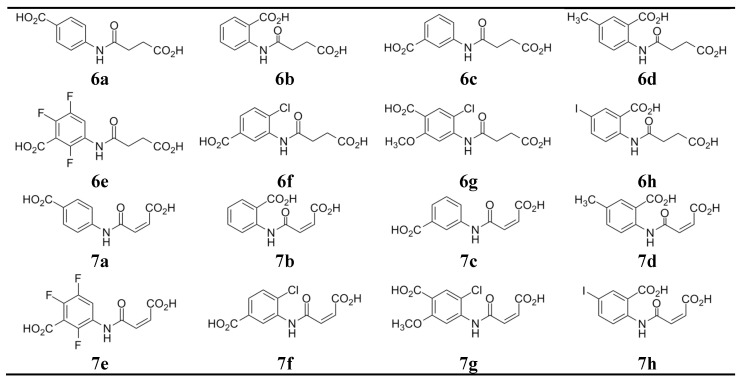
Structures of the tested compounds.

Advances in virtual screening approaches and a better understanding of the 3D structure and molecular determinants of the specific high–affinity interactions between IL–15 and its heterotrimeric receptor complex have led to the discovery of several novel small molecules that effectively reduce IL–15 activity either by interfering with IL–15/IL–2Rβ or γ_c_ [14]. However, despite the appealing advantages of inhibiting IL–15 by selectively blocking IL–15Rα, there has been little progress in developing small molecule inhibitors of this receptor subunit. In the attempt to design highly active IL–15Rα inhibitors, we based our approach on recognition of features characteristic of currently known active and inactive IL–15Rα inhibitors. As shown in Table 1, putative IL–15Rα inhibitors (**R11**–**R16**) are the benzoic acid derivatives, which contain an amide bond in the side chain due to acylation of the amino group with dicarboxylic acids, such as succinic (**R15**, **R16**), maleic (**R12**), 1,2–cyclohexanedicarboxylic (**R11**) and 2,3–norbornanedicarboxylic (**R13**) acids. We aimed to generate non–chiral compounds to simplify analytical procedures and avoid potential issues with their synthesis and purification. Structures of the newly synthesized compounds are presented in Figure 2.

The free energy of binding (Δ*G*_bind_) values for the new molecules were within the range of −11.9 and −14.6 kcal/mol which indicated a very strong binding required for drug–like compounds (Table 2).

The highest Δ*G*_bind_ value of –14.60 kcal/mol and the lowest binding constant (*K*_i_) of 19.8 pM were observed for **7a** (Table 2).

**Figure 3:**
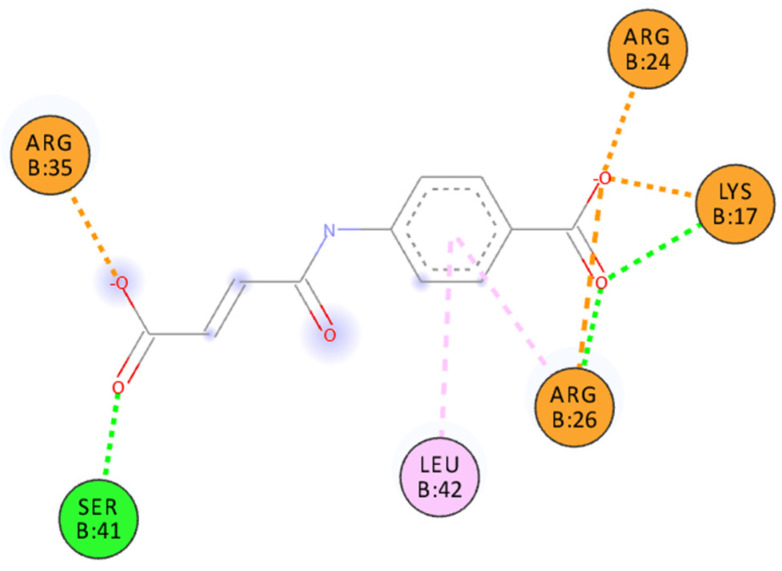
Binding pose of **7a** docked to IL–15Rα. Interactions color codes: green—hydrogen bond, light pink—hydrophobic interaction, orange—charged interaction.

The structure of the IL–15/IL–15Rα complex (PDB code: 2Z3Q) is mainly stabilized with two salt bridges (Arg26:IL–15Rα with Glu53:IL–15 and Arg35:IL–15Rα with Glu46:IL–15) and two strong hydrogen bonds: Arg24:IL–15Rα with the Glu53 backbone oxygen atom (IL–15) and the Arg35 backbone oxygen atom (IL–15Rα) with Tyr26:IL–15. Thus, to interfere with the formation of the IL–15/IL–15Rα complex the potential antagonist should target Arg24, Arg26, and/or Arg35 residues of the receptor (Figure 3). A similar strategy was used previously by us to design a set of ligands with high affinity to IL–15Rα and disrupting IL–15/IL–15Rα binding [11]. As shown in Figure 1 and Figure 3, carboxylic groups of **7a** strongly interact with Arg24/Arg26/Arg35 residues of IL–15Rα and may destabilize IL–15/IL–15Rα complex by hindering interactions between Arg26 from IL–15Rα and E53 from IL–15. The binding poses of the remaining new compounds are presented in the Supplementary Information (Figures S1–S16). Interestingly, we predict that all of the studied compounds bind to at least two of the three crucial Arg residues (Arg24, Arg26, or Arg35). Additionally, in some of them, the carboxylic group of ligands makes a strong salt bridge to Lys17, located also close to the IL–15/IL–15Rα interface (Figure 1). Based on these results and the relatively high Gibbs free binding energy values obtained for all investigated compounds we can expect that in each case we should observe at least a moderate disruption of the IL–15/IL–15Rα interactions.

Table 3 presents the main results of the computational ADME prediction. As shown, all new molecules exhibited drug–like properties. Only minor problems were indicated for **7a**, **7e**, and **7h**: no primary metabolites were indicated for these molecules placing them outside of the range of 95% of drugs. Furthermore, the low values of predicted Caco–2 cell permeability identified for all of the molecules gave rise to one Jorgensen’s rule of three violation and indicated their possible low oral availability.

Interestingly, six of the new putative IL–15Rα inhibitors were previously described in the scientific literature. Compounds **6a**, **6b**, **6c,** and **7a**, **7b**, **7c** were used to develop the quantitative structure–activity relationship model of acetylcholinesterase inhibitors as potential drugs for Alzheimer’s disease [16]. Compounds **6a**, **6b**, and **6c** were also tested as potential antibacterial and antifungal agents [17,18], while **7a**, **7b**, and **7c** were shown to express strong activity as human carbonic anhydrase isoenzymes I and II (hCA I and hCA II) inhibitors [19]. Based on virtual screening, **6b** was identified as an inhibitor of the oncogenic fusion protein RUNX1/ETO [20], a fusion protein comprising leukemia–initiating transcription factor that interferes with the RUNX1 function [21], and a weak inhibitor of the SH2 domain of the tyrosine kinase P56 LCK [22].

Among the new compounds described in this study, close to 90% similarity to known drugs were detected for **6a**, which appeared to be the closest to proxibarbital, a barbiturate derivative ((*RS*)–5–allyl–5–(2–hydroxypropyl)pyrimidine–2,4,6(1*H*,3*H*,5*H*)–trione, CAS: 2537–29–3), for **7a**, which was found close to fosfosal, a derivative of salicylic acid (2–(phosphonooxy)benzoic acid, CAS: 6064–83–1) and for **6c**, which is related to methocarbamol, a carbamate derivative of guaifenesin ((*RS*)–2–hydroxy–3–(2–methoxyphenoxy)propyl carbamate, CAS: 532–03–6).

### 2.3. The Effect of Novel Benzoic Acid Derivatives on PBMC Viability, IL–15–Dependent PBMC Proliferation and TNF–α and IL–17 Release

All cell types constituting peripheral blood mononuclear cells (PBMC), i.e., B cells (~15%), T cells (~70%), monocytes (~5%), and natural killer (NK) cells (~10%), respond to IL–15. Thus, we used PBMC to assess the biological effectiveness of novel benzoic acid derivatives as potential IL–15 inhibitors. To exclude non–specific effects linked to cell death, all of the synthesized compounds were first screened for their cytotoxicity. No cytotoxic effect was observed for evaluated molecules up to 5 mM (data not shown). In all further experiments, the new compounds were used at 5 µM concentration.

**Figure 4:**
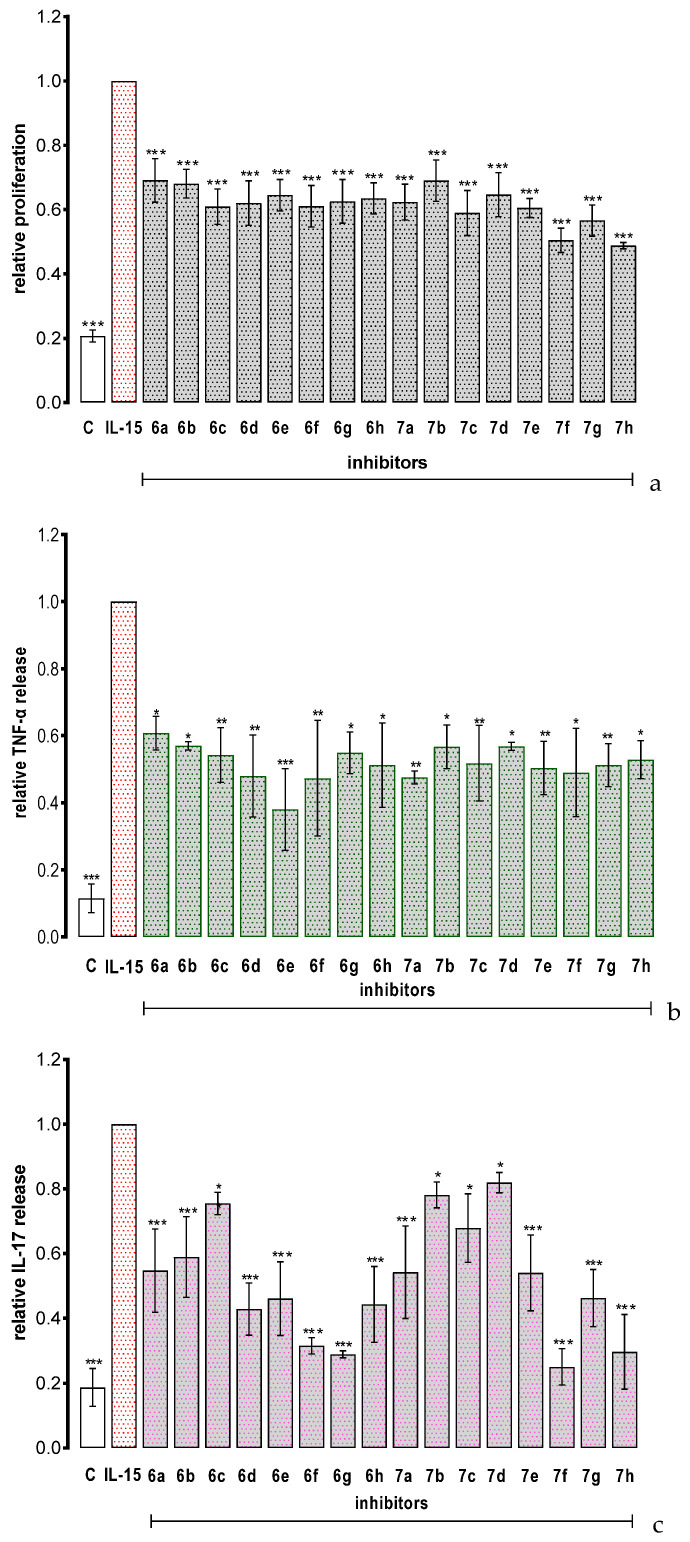
The effect of the tested compounds on IL–15–triggered PBMC response: proliferation (**a**), TNF–α release (**b**) and IL–17 release (**c**). Results are expressed as mean ± SEM of three experiments. Data were analyzed by one way analyses of variance (ANOVA) followed by the post hoc Dunnett test. * *p* < 0.05, ** *p* < 0.01, *** *p* < 0.001 vs. IL–15.

PBMCs do not proliferate spontaneously in vitro, but they divide and release TNF–α and IL–17 in response to IL–15 stimulation [4]. As shown in Figure 4, all the tested compounds significantly inhibited IL–15 depended cell proliferation and strongly inhibited IL–15–dependent PBMC release of TNF–α and IL–17, respectively. The new molecules exerted their biological activity at 5 µM concentration which is significantly lower as compared to the previously reported benzoic acid derivatives, for which the active concentrations were in the range of 50–200 µM (Table 1A) [11]. Importantly, the observed inhibitory effect was superior also to cefazolin, a derivative of 7–aminocephalosporanic acid and a first–generation cephalosporin antibiotic cefazolin which was identified as a small–molecule inhibitor of IL–15Rα and showed promising potential in human proof–of–concept study as a repurposing drug candidate for psoriasis therapy [23]. In vitro, cefazolin reduced IL–15– dependent TNF–α and IL–17 synthesis at 50 µM and 300 µM concentrations, respectively.

## 3. Materials and Methods

### 3.1. Computational Methods

The crystal structure (PDB code: 2Z3Q [12]) of the IL–15/IL–15Rα has been prepared by removing the IL–15 cytokine and water molecules (if present) and adding hydrogen atoms with AutoDockTools 4 [24]. Atomic interaction energy grids were calculated using probes corresponding to each atomic type found in the ligand, at 0.375 Å grid resolution. We have used four different boxes: a 126 Å cubic box centered on the protein and including the entire receptor, and three smaller boxes (50 × 80 × 40 Å, 40 × 70 × 30 Å and 30 × 60 × 30 Å) centered on the protein–protein binding region. In the docking part, we have used Autodock 4.2 [24] with the Genetic Lamarckian Algorithm and standard options, including 100 dockings per compound and 5,000,000 energy evaluations per docking [25]. In all docking experiments, each ligand has been treated in a fully flexible manner with the Gasteiger partial charges added by AutoDockTools 4. In the first phase of docking, the protein has been treated as a completely rigid model, also with Gasteiger partial charges. In the second phase of docking, after finding the likely docking region the protein has been treated as a rigid model, but with four residues (Lys17, Arg24, Arg26, and Arg35) described in a fully flexible manner. For each study’s compound, we have estimated the free energy of binding as well as the inhibition constant *K*_i_ (at 298.15 K) using the approach implemented in Autodock, which uses semiempirical force field to evaluate the sum of differences in energies between unbound and bound states of the ligand. All 2D figures of binding sites were prepared using Discovery Studio Visualizer (Dassault Systemes, 2015). ADME properties were computationally modeled using QikProp ver. 4.6 software (Schrodinger Inc., New York, NY, USA) using default settings.

### 3.2. Compound Synthesis

Fine chemicals and solvents were purchased from commercially available vendors and were used without further purification. Methyl 4–chloro–4–oxobutanoate (**4**) and methyl 4–chloro–4–oxo–2(*Z*)–butenoate (**5**) were obtained according to the literature procedures [26,27]. TLC analyses were performed on plates precoated with silica gel (Merck 60 F_254_, 0.25 mm). HPLC analyses were performed on a Waters system equipped with a LiChrosphere^®^ 100 RP–8 HPLC column and PDA 2996 detector (190–400 nm, 1.2 nm) using ACN:0.1% TFA in H_2_O (50/50 *v*/*v*) as a mobile phase. Samples were dissolved in the mobile phase at a concentration of 0.1 mg/mL (**7c** and **7f**) or 0.25 mg/mL (the remaining compounds). Analyses were performed at the wavelengths given for each separately described compound. Melting points were determined throughout DSC measurements (DSC822e cell) with an IntraCooler (Mettler Toledo GmbH, Gießen, Germany) in the nitrogen atmosphere. Samples (5–10 mg used as received) in a standard (40 μL) aluminum pan were heated from 25 to 300 °C (10 °C/min). IR spectra were recorded in a KBr pellet containing ca 1 mg of the tested compound and ca 200 mg of KBr using Nicolet iS10 spectrometer in the range of 4000–400 cm^−1^ (resolution 4 cm^−1^). NMR spectra were recorded on a Varian VNMRS–600 spectrometer in 25 °C for DMSO–*d*_6_ solutions using TMS as the internal standard in the following ranges (δ ppm): 0–14 (^1^H), 20–180 (^13^C) and –146 to –124 (^19^F); chemical shifts (δ) are quoted in ppm and coupling constants (*J*) in Hz. Mass spectrometry (MS and HRMS) was carried out using AutoSpec Premier (Waters) spectrometer with EI ionization. Absorbance measurements in colorimetric in vitro tests were performed using SPECTROstar Nano spectrometer at the wavelength suggested by tests manufacturers.

#### 3.2.1. Synthesis of Succinic Derivatives 

**Scheme 1:**
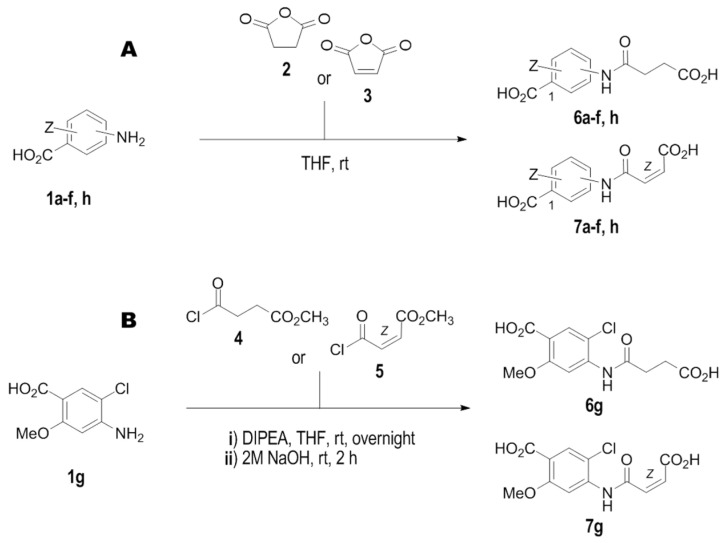
The schematic representation of synthesis of novel IL–15Rα inhibitors. (**A**). For compounds **6a**–**f**, **h**, and **7a**–**f**, **h**. (**B**). For compounds **6g** and **7g**. Conditions: (i) THF, rt; (ii) DIPEA, THF, 50 °C then 2 M aq. NaOH, rt.

Synthesis of compounds **6a**–**6f** and **6h** was performed according to Scheme 1A.

4–[(3–carboxy–1–oxopropyl)amino]benzoic acid (**6a**):

1.75 g (12.8 mmol) of 4–aminobenzoic acid (**1a**) is dissolved in 20 mL of THF obtaining a clear solution (**I**). Separately, 1.73 g of succinic anhydride (**2**) is dissolved in 25 mL of THF resulting clear, colorless solution (**II**). Solution II is added to a well stirred solution I and the whole mixture is stirred for 1 h at rt. The precipitated solid is separated, washed with cold THF, and dried on air to give 2.64 g (11.1 mmol) of the expected product as a white powder. **Purity**: 99.93% (HPLC at 254 nm). **Yield**: 87%. **Mp**.: 234.9 °C (lit. Mp.: 225 °C [28]). **IR**: 3308 (NH and OH), 3100–3000 (CH_arom._), 2928 (CH_aliph_), 2655–2552 (chelat C=O…HO), 1698–1664 (C=O), 1608–1595 (C=C), 1529 (C(O)–NH). **^1^H NMR**: 12.30 (bs, ≈1.5H) 2 × CO_2_H, 10.26 (s, 1H) NH, 7.88 (d, *J*_H2/H6–H3/H5_ = 8.70 Hz, 2H) H2/H6′, 7.70 (d, *J*_H3/H5–H2/H6_ = 8.70 Hz, 2H) H3/H5′, 2.60 (t, *J* = 6.6 Hz, 2H) COCH_2_, 2.53 (t, *J* = 6.6 Hz, 2H) CH_2_CO_2_H. **^13^C NMR**: 174.00 (C_aliph_O_2_H), 170.91 (C=O), 167.16 (C_arom._O_2_H), 143.45 (C4), 130.56 (C2C6), 125.04 (C1), 118.35 (C3C5), 31.34 (COCH_2_), 28.84 (C8). **HRMS**: calc. for C_11_H_10_NO_5_: 236.0559; found: 236.0559.

Succinic derivatives **6b**–**f** and **6h** were obtained according to the same protocol, using appropriate aminobenzoic acid (**1b**–**f**, **h**) and succinic anhydride (**2**).

2–[(3–carboxy–1–oxopropyl)amino]benzoic acid (**6b**):

White powder. **Purity**: 99.88% (HPLC at 254 nm). **Yield**: 56%. **Mp**.: 187.9 °C (lit. Mp.: 184 °C [28], 188 °C [29], 170 °C [30]). **IR**: 3322 (NH and OH), 3100–3000 (CH_arom._), 2938 (CH_aliph_), 2659 (chelate C=O…HO), 1683 (C=O), 1598–1586 (C=C), 1528 (C(O)–NH). **^1^H NMR**: 13.46 (bs, 1H) CO_2_H_aliph_, 12.38 (bs, 1H) CO_2_H_arom._, 11.17 (s, 1H) NH, 8.49 (d, *J*_H3–H4_ = 8.4 Hz, 1H) H3, 7.99 (dd, *J*_H6–H5_ = 7.9 Hz, *J*_H6–H4_ = 1.55 Hz, 1H) H6, 7.59 (ddd, *J*_H4–H5_ = 7.8 Hz, *J*_H4–H6_ = 1.55 Hz, 1H) H4, 7.15 (ddd, J = 7.6 Hz, *J*_H5–H3_ = 0.93 Hz, 1H) H5, 2.64 (t, *J* = 6.6 Hz, 2H) COCH_2_, 2.57 (t, *J* = 6.6 Hz, 2H) CH_2_CO_2_H. **^13^C NMR**: 173.60 (C_aliph_O_2_H), 170.23 (C=O), 169.56 (C_arom._O_2_H), 140.84 (C2), 134.08 (C4), 131.10 (C6), 122.49 (C5), 119.79 (C3), 116.19 (C1), 38.14 (COCH_2_), 28.68 (CH_2_CO_2_H). **HRMS**: calc. for C_11_H_10_NO_5_: 236.0559; found: 236.0560.

3–[(3–carboxy–1–oxopropyl)amino]benzoic acid (**6c**):

White powder. **Purity**: 99.87% (HPLC at 240 nm). **Yield**: 97%. **Mp**.: 212.0 °C (lit. Mp.: 221–222 °C [28], 236–238 °C [31,32]). **IR**: 3293 (NH and OH), 3081 (CH_arom._), 2924 (CH_aliph_), 2626 (chelate C=O…HO), 1694–1660 (C=O), 1593 (C=C). 1545 C(O)–NH). **^1^H NMR**: 12.56 (bs, ≈2H) 2 × CO_2_H, 10.17 (s, 1H) NH, 8.30 (s, 1H) H2, 7.85 (dd, 1H) H4, 7.65 (d, 1H) H6, 7.44 (t, 1H) H5, 2.63 (t, *J* = 6.0 Hz, 2H) COCH_2_, 2.59 (t, *J* = 6.0 Hz, 2H) CH_2_CO_2_H. **^13^C NMR**: 174.01 (C_aliph_O_2_H), 170.58 (C=O), 167.40 (C_arom._O_2_H), 139.62 (C3), 131.42 (C1), 129.08 (C5), 123.97 (C6), 123.20 (C4), 119.90 (C2), 31.21 (COCH_2_), 28.89 (CH_2_CO_2_H). **HRMS**: calc. for C_11_H_10_NO_5_: 236.0559; found: 236.0559.

2–[(3–carboxy–1–oxopropyl)amino]–5–methylbenzoic acid (**6d**):

Off–white powder. **Purity**: 99.49% (HPLC at 240 nm). **Yield**: 40%. **Mp**.: 177.0 °C. **IR**: 3388, 3338 (NH and OH), 3100–3000 (CH_arom._), 2960 (CH_aliph_), 2563 (chelate C=O…HO), 1686 (C=O), 1593 (C=C). 1523 (C(O)–NH). **^1^H NMR**: 12.78 (bs, 2H) 2 × CO_2_H, 11.04 (s, 1H) NH, 8.36 (d, *J*_H3–H4_ = 8.5 Hz, 1H) H3, 7.78 (d, *J*_H6–H4_ = 2.0 Hz, 1H) H6, 7.38 (dd, *J*_H4–H3_ =8.5 Hz, *J*_H4–H6_ = 2.0 Hz, 1H) H4, 2.61 (t, *J* = 6.60 Hz, 2H) COCH_2_, 2.54 (t, *J* = 6.60 Hz, 2H) CH_2_CO_2_H, 2.29 (s, 3H) CH_3_. **^13^C NMR**: 173.61 (C_aliph_O_2_H), 169.97 (C=O), 169.56 (C_arom._O_2_H), 138.51 (C2), 134.59 (C4), 131.53 (C5), 131.08 (C6), 119.89 (C3), 116.19 (C1), 32.06 (COCH_2_), 28.71 (CH_2_CO_2_H), 20.16 (CH_3_). **HRMS**: calc. for C_12_H_12_NO_5_: 250.0715; found: 250.0718.

3–[(3–carboxy–1–oxopropyl)amino]–2,5,6–trifluorobenzoic acid (**6e**):

Off–white powder. **Purity**: 99.85% (HPLC at 248 nm). **Yield**: 66%. **Mp**.: 161.3 °C. **IR**: 3319 (NH and OH), 3100–3000 (CH_arom._) 2934 (CH_aliph_), 2656–1551 (chelate C=O…HO), 1694 (C=O), 1546 (C=C and C(O)–NH). **^1^H NMR**: 12.20 (bs, 2H) 2 × CO_2_H, 11.06 (s, 1H) NH, 8.14 (d, *J*_H–F5_ = 8.3 Hz, 1H) H_arom._, 2.65 (t, 2H) COCH_2_, 2.52 (t, 2H) CH_2_CO_2_H. **^13^C NMR**: 173.69 (C_aliph_O_2_H), 171.18 (C=O), 161.07 (C_arom._O_2_H), 145.84 (C2), 145.24 (C6), 142.44 (C5), 123.53 (C3), 113.58 (C1), 112.90 (C4), 30.64 (COCH_2_), 28.64 (CH_2_CO_2_H). **^19^F NMR**: –142.90 (dd, *J*_F5–F6_ = 24.0 Hz, *J*_F5–H_ = 8.3 Hz, 1F) F5, –140.70 (dd, *J*_F6–F5_ = 24.0 Hz, *J*_F6–F2_ = 13.0 Hz, 1F) F6, –128.10 (dd, *J*_F2–F6_ = 13.0 Hz, 1F) F2. **HRMS**: calc. for C_11_H_8_NO_5_F_3_: 291.0349; found: 291.0355.

3–[(3–carboxy–1–oxopropyl)amino]–4–chlorobenzoic acid (**6f**):

White powder. **Purity**: 98.05% (HPLC at 254 nm). **Yield**: 65%. **Mp**.: 241.9 °C. **IR**: 3279 (NH and OH), 3056 (CH_arom._) 2969 (CH_aliph_), 2644 (chelat C=O…HO), 1698–1675 (C=O), 1598–1580 (C=C), 1537 (C(O)–NH). **^1^H NMR**: 12.69 (bs, 2H) 2 × CO_2_H, 9.67 (s, 1H) NH, 8.38 (s, 1H) H2, 7.72 (d, *J* = 8.4 Hz, 1H) H6, 7.61 (d, *J* = 8.4 Hz, 1H) H5, 2.71 (t, *J* = 6.0 Hz, 2H) COCH_2_, 2.58 (t, *J* = 6.0 Hz, 2H) CH_2_CO_2_H. **^13^C NMR**: 173.88 (C_aliph_O_2_H), 170.95 (C=O), 166.49 (C_arom._O_2_H), 135.29 (C3), 130.44 (C4), 130.03 (C1), 129.82 (C5), 126.49 (C6), 126.40 (C2), 30.74 (COCH_2_), 28.95 (CH_2_CO_2_H). **HRMS**: calc. for C_11_H_9_NO_5_Cl: 270.0169; found: 270.0165.

2–[(3–carboxy–1–oxopropyl)amino]–5–iodobenzoic acid (**6h**):

White powder. **Purity**: 99.37% (HPLC at 254 nm). **Yield**: 96%. **Mp**.: 217.5 °C. **IR:** 3340 (NH and OH), 3100–3000 (CH_arom._), 2942 (CH_aliph_), 2611 (chelat C=O…HO), 1695–1671 (C=O), 1589–1570 (C=C), 1507 (C(O)–NH). **^1^H NMR**: 13.80 (bs, 1H) CO_2_H_aliph_, 12.33 (bs, 1H) CO_2_H_arom._, 11.07 (s, 1H) NH, 8.28 (d, *J*_H3–H4_ = 8.80 Hz, 1H) H3, 8.22 (d, *J*_H6–H4_ = 2.20 Hz, 1H) H6, 7.90 (dd, *J*_H4–H3_ = 8.80 Hz, *J*_H4–H6_ = 2.20 Hz, 1H) H4, 2.63 (t, *J* = 6.60 Hz, 2H) COCH_2_, 2.55 (t, *J* = 6.60 Hz, 2H) CH_2_CO_2_H. **^13^C NMR**: 173.53 (C_aliph_O_2_H), 170.37 (C=O), 168.15 (C_arom._O_2_H), 142.22 (C4), 140.36 (C2), 138.95 (C6), 122.03 (C3), 118.52 (C1), 85.57 (C5), 32.14 (COCH_2_), 28.61 (CH_2_CO_2_H). **HRMS**: calc. for C_11_H_10_NO_5_Ina: 385.9501; found: 385.9495.

Synthesis of the compound **6g** was performed according to Scheme 1B.

4–[(3–carboxy–1–oxopropyl)amino]–2–methoxy–5–chlorobenzoic acid (**6g**):

2.02 g (10 mmol) of 2–methoxy–4–amino–5–chlorobenzoic acid (**1g**) is dissolved in 60 mL of THF. 2.5 mL (20 mmol) of methyl 4–chloro–4–oxobutanoate (**4**) is added dropwise to this solution followed by the addition of 3.4 mL (20 mmol) of DIPEA and the mixture is stirred overnight. The solution is filtered through celite and then evaporated to obtain yellowish oil. 30 mL of 2 M NaOH is then added to this oil and the solution is stirred at rt until TLC analysis shows the hydrolysis completion (ca 2 h). The mixture is then acidified with 2 M HCl to pH 4 and the suspension is left at 5 °C overnight. The precipitated solid is filtered off, washed with water and hot methanol, and dried on air to obtain 2.46 g (8.15 mmol) of the expected product as a white powder. **Purity**: 96.35% (HPLC at 278 nm). **Yield**: 82%. **Mp**.: 223.5 °C. **IR**: 3527, 3403 (NH and OH), 3100–3000 (CH_arom._), 2939–2984 (CH_aliph_), 1698 (C=O), 1624–1585 (C=C), 1522 (C(O)–NH), 1089 (C–O). **^1^H NMR**: 9.64 (s, 1H) NH, 7.66 (s, 1H) H3, 7.55 (s, 1H) H6, 3.74 (s, 1H) CH_3_, 2.67 (t, *J* = 6.6 Hz, 2H) COCH_2_, 2.51 (t, *J* = 6.6 Hz, 2H) CH_2_CO_2_H. **^13^C NMR**: 174.08 (C_aliph_O_2_H), 171.19 (C=O), 166.90 (C_arom._O_2_H), 156.45 (C2), 137.13 (C4), 130.37 (C6), 122.74 (C1), 115.13 (C5), 108.12 (C3), 55.86 (OCH_3_), 31.32 (COCH_2_), 29.41 (CH_2_CO_2_H). **HRMS**: calc. for C_12_H_11_NO_6_Cl: 300.0275; found: 300.0276.

#### 3.2.2. Synthesis of Maleic Derivatives

Synthesis of **7a**–**7f** and **7h** was performed according to the Scheme 1A.

4–[(2*Z*)–3–carboxy–1–oxo–2–propen–1–yl]aminobenzoic acid (**7a**):

1.66 g (12.1 mmol) of (**1a**) is dissolved in 15 mL of THF obtaining a clear solution (**I**). Separately, 1.30 g (13.3 mmol) of maleic anhydride (**4**) is dissolved in 20 mL of THF obtaining a clear, colorless solution (**II**). Solution **II** is added to a well stirred solution **I** and the whole is stirred for 1 h at rt. The precipitated solid is separated, washed with cold THF, and dried on air to give 1.96 g (8.33 mmol) of the expected product as a light–yellow powder. **Purity**: 99.58% (HPLC at 278 nm). **Yield**: 69%. **Mp**.: 195.2 °C (lit. Mp.: 252 °C [33], 242–244 °C [34], 218–220 °C [35,36], 215–216 °C [37], 210 °C [38], 224 °C [32]). **IR**: 3315 (NH and OH), 3100–3000 (CH_arom._ and C=C), 2880 (CH_aliph_), 2667–2541 (chelat C=O…HO), 1694 (C=O), 1609–1542 (C=C and C(O)–NH). **^1^H NMR**: 12.83 (bs, ≈1.5H) 2 × CO_2_H, 10.60 (s, 1H) NH, 7.92 (dd, *J*_H2–H5_ = *J*_H6–H3_ = 1.83 Hz, *J*_H2/H6–H3/H5_ = 8.80 Hz, 2H) H2/H6′, 7.74 (d, *J*_H3/H5–H2H6_ = 8.80 Hz, 2H) H3/H5, 6.50 (d, *J* = 12.11 Hz, 1H) COCH=CHCO_2_H, 6.33 (d, *J* = 12.11 Hz, 1H) COCH=CHCO_2_H. **^13^C NMR**: 167.08 (C_aliph_O_2_H), 167.06 (C_arom._O_2_H), 163.83 (C=O), 142.84 (C4), 131.87 (COCH=CH), 130.58 (C2C6), 130.35 (CH=CHCO_2_H), 125.74 (C1), 118.90 (C3C5). **HRMS**: calc. for C_11_H_8_NO_5_: 234.0402; found: 234.0401.

Following the same manner, maleic derivatives (**7b**–**f**, **h**) were obtained using appropriate aminobenzoic acid (**1b**–**f**, **h**) and maleic anhydride (**3**).

2–[(2*Z*)–3–carboxy–1–oxo–2–propen–1–yl]aminobenzoic acid (**7b**):

Off–white powder. **Purity**: 99.64% (HPLC at 310 nm). **Yield**: 59%. **Mp**.: 190.3 °C (lit. Mp.: 277 °C [39], 205 °C, 228 °C (dec) [33], 192 °C [40], 158 °C [30]). **IR:** 3300–3000 (NH and OH and CH_arom._ and CH_aliph_), 2607–2401 (chelat C=O…HO), 1694 (C=O), 1615–1578 (C=C). 1534 (C(O)–NH). **^1^H NMR**: ≈13.30 (bs, 2H) 2 × CO_2_H, 11.33 (s, 1H) NH, 8.48 (d, *J*_H3–H4_ = 8.3 Hz, 1H) H3, 8.00 (dd, *J*_H6–H5_ = 8.0 Hz, *J*_H6–H4_ = 1.7 Hz, 1H) H6, 7.62 (ddd, *J*_H4–H3_ = 8.3 Hz, *J*_H4–H5_ = 7.3 Hz, *J*_H4–H6_ = 1.7 Hz, 1H) H4, 7.20 (ddd, *J*_H5–H6_ = 8.0 Hz, *J*_H5–H4_ = 7.3 Hz, *J*_H5–H3_ = 1.2 Hz, 1H) H5, 6.60 (d, *J* = 12.0 Hz, 1H) COCH=CHCO_2_H, 6.31 (d, *J* = 12.0 Hz, 1H) COCH=CHCO_2_H. **^13^C NMR**: 169.36 (C_arom._O_2_H), 166.48 (C=O), 163.63 (C_aliph_O_2_H), 140.19 (C2), 134.09 (C4), 133.37 (COCH=CH), 131.15 (C6), 129.28 (CH=CHCO_2_H), 123.27 (C5), 120.30 (C3), 116.95 (C1). **HRMS**: calc. for C_11_H_8_NO_5_: 234.0402; found: 234.0405.

3–[(2*Z*)–3–carboxy–1–oxo–2–propen–1–yl]aminobenzoic acid (**7c**):

Off–white powder. **Purity**: 99.90% (HPLC at 278 nm). **Yield**: 59%. **Mp**.: 182.5 °C (lit. Mp.: 245 °C, 280 °C (dec) [33], 218–220 °C [31], 215–217 °C [37]). **IR**: 3286 (NH and OH), 3106 (CH_arom._ and CH_aliph_), 2674 (chelate C=O…HO), 1713 (C=O), 1569 (C=C and C(O)–NH). **^1^H NMR**: 13.06 (bs, ≈1.5H) 2 × CO_2_H, 10.58 (s, 1H) NH, 8.34 (s, 1H) H2, 7.87 (dd, 1H) H4, 7.72 (d, 2H) H6, 7.49 (t, 1H) H5, 6.53 (d, *J* = 12.0 Hz, 1H) COCH=CHCO_2_H, 6.36 (d, *J* = 12.0 Hz, 1H) COCH=CHCO_2_H. **^13^C NMR**: 167.28 (C_arom._O_2_H), 167.05 (C_aliph_O_2_H), 163.69 (C=O), 139.00 (C3), 131.92 (COCH=CH), 131.53 (C1), 130.41 (CH=CHCO_2_H), 129.24 (C5), 134.77 (C6), 123.75 (C4), 120.44 (C2). **HRMS**: calc. for C_11_H_8_NO_5_: 234.0402; found: 234.0402.

2–[(2*Z*)–3–carboxy–1–oxo–2–propen–1–yl]amino–5–methylbenzoic acid (**7d**):

Yellow powder. **Purity**: 99.72% (HPLC at 310 nm). **Yield**: 40%. **Mp**.: 193.6 °C. **IR**: 3443 (NH and OH), 3051 (CH_arom._ and CH_aliph_), 2511 (chelate C=O…HO), 1697–1678 (C=O), 1616–1528 (C=C and C(O)–NH). **^1^H NMR**: ≈13.2 (bs, 2H) 2 × CO_2_H, 11.22 (s, 1H) NH, 8.35 (d, *J*_H3–H4_ = 8.2 Hz, 1H) H3, 7.81 (s, *J*_H6–H4_ = 2.4 Hz, 1H) H6, 7.44 (d, *J*_H4–H3_ = 8.2 Hz, 1H) H4, 6.58 (d, *J* = 12.0 Hz, 1H) COCH=CHCO_2_H, 6.30 (d, *J* = 12.0 Hz, 1H) COCH=CHCO_2_H, 2.31 (s, 1H) CH_3_. **^13^C NMR**: 169.35 (C_aliph_O_2_H), 166.52 (C=O), 163.41 (C_arom._O_2_H), 137.72 (C2), 134.58 (C4), 133.21 (COCH=CH), 132.56 (C5), 131.18 (C6), 129.45 (CH=CHCO_2_H), 120.46 (C3), 117.06 (C1), 20.24 (CH_3_). **HRMS**: calc. for C_12_H_10_NO_5_: 248.0559; found: 248.0559.

3–[(2*Z*)–3–carboxy–1–oxo–2–propen–1–yl]amino–2,5,6–trifluorobenzoic acid (**7e**):

Light–violet powder. **Purity**: 99.65% (HPLC at 254 nm). **Yield**: 54%. **Mp**.: 164.1 °C. **IR**: 3299 (NH and OH), 3100–3000 (CH_arom._ and CH_aliph_), 2881 (CH_aliph_), 2575 (chelat C=O…HO), 1726–1685 (C=O), 1602–1553 (C=C and C(O)–NH). **^1^H NMR**: 12.88 (bs, 2H) 2 × CO_2_H, 10.46 (s, 1H) NH, 8.15 (d, *J*_H–F_ = 7 Hz, 1H) H_arom._, 6.52 (d, *J* = 12.0 Hz, 1H) COCH=CHCO_2_H, 6.35 (d, *J* = 12.0 Hz, 1H) COCH=CHCO_2_H. **^13^C NMR**: 167.31 (C_aliph_O_2_H), 163.90 (C=O), 161.12 (C_arom._O_2_H), 146.37 (C2), 145.49 (C6), 143.17 (C5), 130.95 (CH=CHCO_2_H), 130.59 (COCH=CH), 123.10 (C3), 113.75 (C1), 113.24 (C4). **^19^F NMR**: −141.73 (dd, 1F, *J*_F–H_ = 7.5 Hz, *J*_F5–F6_ = 24 Hz) F5, −140.44 (dd, 1F, *J*_F6–F5_ = 24 Hz) F6, −127.55 (dd, 1F) F2. **HRMS**: calc. for C_11_H_6_NO_5_F_3_: 289.0198; found: 289.0203.

3–[(2*Z*)–3–carboxy–1–oxo–2–propen–1–yl]amino–4–chlorobenzoic acid (**7f**):

Off–white powder. **Purity**: 99.13% (HPLC at 254 nm). **Yield**: 59%. **Mp**.: 184.9 °C. **IR**: 3313 (NH and OH), 3037 (CH_arom._ and CH_aliph_), 2552 (chelate C=O…HO), 1701 (C=O), 1616–1583 (C=C), 1547 (C(O)–NH). **^1^H NMR**: 13.12 (bs, 2H) 2 × CO_2_H, 10.15 (s, 1H) NH, 8.40 (s, 1H) H2, 7.75 (d, *J*_H6–H5_ = 8.4 Hz, *J*_H6–H2_ = 2.0 Hz, 1H) H6, 7.64 (d, *J*_H5–H6_ = 8.4 Hz, 1H) H5, 6.61 (d, *J* = 11.9 Hz, 1H) COCH=CHCO_2_H, 6.37 (d, *J* = 11.9 Hz, 1H) COCH=CHCO_2_H. **^13^C NMR**: 167.08 (C_aliph_O_2_H), 166.30 (C_arom._O_2_H), 163.72 (C=O), 134.62 (C3), 131.22 (COCH=CH), 130.78 (C4), 130.72 (CH=CHCO_2_H), 130.02 (C1), 129.89 (C5), 127.00 (C6), 126.54 (C2). **HRMS**: calc. for C_11_H_7_NO_5_Cl: 268.0013; found: 268.0013.

2–[(2*Z*)–3–carboxy–1–oxo–2–propen–1–yl]amino–5–iodobenzoic acid (**7h**):

Yellow powder. **Purity**: 99.53% (HPLC at 278 nm). **Yield**: 82%. **Mp**.: 181.7 °C (dec). **IR**: 3431 (NH and OH), 3084 (CH_arom._ and CH_aliph_), 2475 (chelate C=O…HO), 1697 (C=O), 1621–1514 (C=C and C(O)–NH). **^1^H NMR**: ≈13.80 (bs, 1H) CO_2_H_aliph_, ≈13.00 (bs, 1H) CO_2_H_arom._, 11.22 (s, 1H) NH, 8.28 (d, *J*_H3–H4_ = 8.86 Hz, 1H) H3, 8.24 (d, *J*_H6–H4_ = 2.20 Hz, 1H) H6, 7.94 (dd, *J*_H4–H3_ = 8.86, *J*_H4–H6_ = 2.20 Hz, 1H) H4, 6.59 (d, *J* = 11.9 Hz, 1H) COCH=CHCO_2_H, 6.32 (d, *J* = 11.9 Hz, 1H) COCH=CHCO_2_H. **^13^C NMR**: 167.99 (C_arom_O_2_H), 166.48 (C_aliph_O_2_H), 163.65 (C=O), 142.29 (C4), 139.75 (C2), 139.03 (C6), 133.09 (COCH=CH), 129.49 (CH=CHCO_2_H), 122.42 (C3), 119.13 (C1), 86.58 (C5). **HRMS**: calc. for C_11_H_7_NO_5_I: 359.9369; found: 359.9373.

Synthesis of **7g** was performed according to the Scheme 1B.

4–[(2*Z*)–3–carboxy–1–oxo–2–propen–1–yl]amino–2–methoxy–5–chlorobenzoic acid (**7g**):

2.02 g (10 mmol) of 2–methoxy–4–amino–5–chlorobenzoic acid (**1g**) is dissolved in 60 mL of THF. 1.7 mL (15 mmol) of methyl 4–chloro–4–oxo–2–(*Z*)–butenoate (**5**) is added dropwise to this solution followed by the addition of 2.9 mL (17 mmol) of DIPEA and the mixture is stirred overnight. The solution is filtered through celite and then evaporated to obtain brown oil. This oily product is dissolved in 20 mL of ethyl acetate and 20 mL of water is added. The white precipitate is filtered, washed twice with water, and placed into the flask. Ten ml of 2 M NaOH is added and suspension is stirred for 2 h, than whole is acidified with 2 M HCl to pH 4. The solid is filtered off, washed with cold water, and dried on air to obtain 2.38 g (7.9 mmol) of the expected product as a yellowish powder. **Purity**: 94.56% (HPLC at 278 nm). **Yield**: 79%. **Mp**.: 250.4 °C. **IR**: 3544, 3378, 3266 (NH and OH), 3084 (CH_arom._ and CH_aliph_), 2594 (chelat C=O…HO), 1732–1683 (C=O), 1585 (C=C), 1530 (C(O)–NH). **^1^H NMR**: ≈12.89 (bs, 2H) 2 × CO_2_H, 10.17 (s, 1H) NH, 7.85 (s, 1H) H3, 7.76 (s, 1H) H6, 7.45 (d, *J* = 15.4 Hz, 1H) COCH=CHCO_2_H, 6.71 (d, *J* = 15.4 Hz) COCH=CHCO_2_H. **^13^C NMR**: 166.23 (C_aliph_O_2_H), 165.43 (C_arom._O_2_H), 162.60 (C=O), 157.46 (C2), 138.38 (C4), 136.41 (COCH=CH), 131.98 (CH=CHCO_2_H), 131.41 (C6), 118.40 (C1), 115.77 (C5), 108.70 (C3), 56.14 (OCH_3_). **HRMS**: calc. for C_12_H_9_NO_6_Cl: 298.0118; found: 298.0117.

### 3.3. Preparation of PBMC

PBMCs were isolated from buffy coats of healthy male donors aged 25–35 years (Warsaw Blood Donation Centre, Poland). Thirty ml of blood twice diluted in 0.9% NaCl was layered on 15 mL of Lymphoprep (Axis–shield) and centrifuged at 800× *g* for 15 min. The layer of PBMC was collected, and cells were washed twice in 0.9% NaCl and suspended in RPMI 1640 medium (Gibco/Thermo Fisher Scientific, New York, NY, USA), containing 10 mM HEPES (Sigma-Aldrich Polska, Poznań, Poland), 2% fetal bovine serum (Biowest) and 1% antibiotic–antimycotic solution (streptomycin sulfate, sodium penicillate G, amphotericin B) (PAA) and seeded for the experiment.

### 3.4. Cytotoxicity and Cell Proliferation Assays

Cytotoxicity of the compounds tested was assessed by determining the amount of LDH released from cells using the CytoTox 96 Non–Radioactive Cytotoxicity Assay (Promega, Madison, WI, USA) according to the manufacturer’s instructions. None of the tested substances showed cytotoxicity up to 5 mM (data not shown).

The effect of the investigated compounds on IL–15 dependent PBMCs proliferation was assessed by determining DNA synthesis in replicating cells with the BrdU Cell Proliferation Assay (Calbiochem, Merck Group) according to the manufacturer’s instructions. Briefly, freshly isolated PBMCs were seeded in a 96–well V–bottom plate (25 × 10^3^ cells in 100 µL of the culture medium/well) Next, the cells were incubated for 30 min with the tested compounds at 5 µM concentrations. Next, 10 ng/mL IL–15 (R&D Systems, Minneapolis, MN, USA) was added and cells were incubated for 72 h at 37 °C, 5% CO_2_. For the last 24 h of incubation, bromodeoxyuridine (BrdU) was added to the culture medium at the concentration recommended by the manufacturer. Then, the cells were centrifuged (160× *g*, 10 min) and fixed. Further experimental steps were performed according to the manufacturer’s protocol. The experiment was performed using cells isolated from three blood donors, each in five technical replicates.

### 3.5. TNF–α and IL–17 Secretion

Freshly isolated PBMCs were seeded in a 48–well plate (1 × 10^6^ cells in 0.5 mL of the culture medium per well) and treated with the tested compound at 5 µM concentrations for 30 min and then, 10 ng/mL IL–15 (R&D Systems, Minneapolis, MN, USA) was added for 48 h. Next, the culture media from each well were collected, centrifuged (10,000× *g*, 10 min, 4 °C), and frozen at −80 °C until the level of cytokine was measured using ELISA tests. Cells were harvested, lysed in 0.1 M NaOH, and frozen at −80 °C until the total protein level measurement. Each experiment was performed using cells isolated from three blood donors, each in two technical replicates.

Total protein levels were measured in PBMCs using the BCA Protein Assay Kit (Pierce Biotechnology, Waltham, MA, USA). The concentrations of IL–17 and TNF–α were measured in culture supernatants of cytokine–stimulated cells using the Human TNF–α Quantikine ELISA Kit (R&D Systems, Minneapolis, MN, USA) and Human IL–17A High Sensitivity ELISA (eBioscience, San Diego, CA, USA), respectively, according to the manufacturer’s instructions. The values obtained for IL–17 and TNF–α concentrations were calculated per 1 mg of total protein.

### 3.6. Statistical Analysis

Statistical significance was assessed by ANOVA with the Dunnett *post hoc* test. *p* values below 0.05 were considered statistically significant. Statistical analyses were performed using GraphPad Prism 9.3.1 software (GraphPad Software). Data were presented as the mean ± SEM from at least three independent experiments.

## 4. Conclusions

Abnormal expression or dysregulated IL–15 signaling plays a key role in the pathogenic development of numerous autoimmune and inflammatory diseases. First positive proof–of–concept for the clinical effects of topical cefazolin, first–generation cephalosporin which was identified as a small molecule IL–15Rα inhibitor and used in the treatment of psoriasis confirms this approach [23]. In the present study, we determined the structure activity relationship of benzoic acid derivatives representing the predominant subset of the currently known IL–15Rα inhibitors and identified the structural requirements necessary for the anti–IL–15 activity. This allowed us to design a series of 16 new compounds, all of which demonstrated a superior inhibitory effect on IL–15 dependent responses in vitro. The rational design of IL–15 inhibitors may propel the identification of potential lead molecules for the development of safe and effective therapeutic agents.

## Figures and Tables

**Table 1:** Benzoic acid derivatives identified previously through in silico modelling and pharmacophores screening as putative small molecule IL–15Rα inhibitors [11]. **A.** Molecules with biological activity proven at the indicated concentrations. **B.** Inactive benzoic acid derivatives.

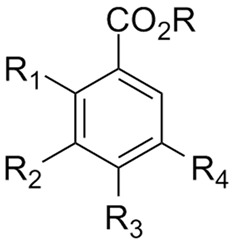					
**A.**					
**Chemical Name (Compound)** **Active Concentration**	**R**	**R_1_**	**R_2_**	**R_3_**	**R_4_**
4–{[1–(4–fluorophenyl)–1(*H*)–tetrazol–5–yl]thio}methyl-benzoic acid (**R9**) 50 μM	H	H	H	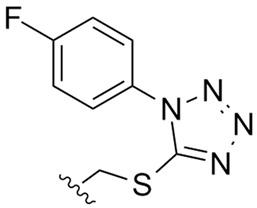	H
methyl 2,4-dihydroxy–3–methylbenzoate (**R10**) 50 μM	CH_3_	OH	CH_3_	OH	H
4–{[(2–carboxycyclohexyl)- carbonyl]amino}methyl-benzoic acid (**R11**) 100 μM	H	H	H	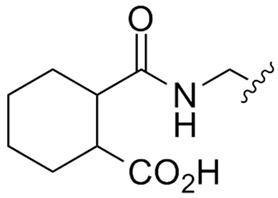	H
3–[(2–carboxyethenyl)amino]–5–[(3–carboxy–1–oxo–2–propen–1–yl)amino]benzoic acid (**R12**) 200 μM	H	H	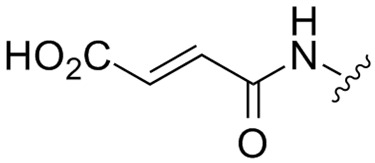	H	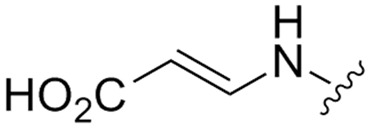
4–{[2–carboxy(bicyclo [2.2.1]hept–1–yl)carbonyl]amino}- methylbenzoic acid (**R13**) 200 μM	H	H	H	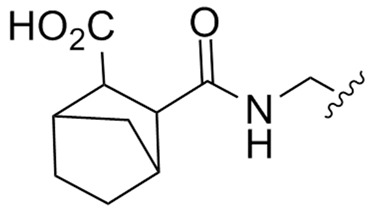	H
3,5–bis–[(3–carboxy–1–oxo– propyl)amino]benzoic acid (**R15**) 100 μM	H	H	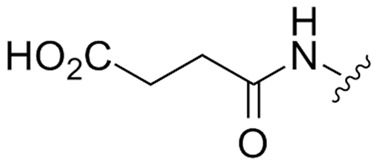	H	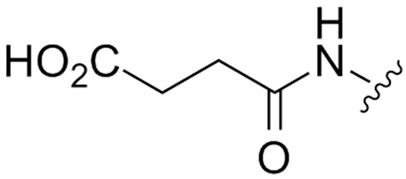
4–{[(4–methoxy–1,4–dioxobutyl)- amino]methyl}benzoate (**R16**) 100 μM	CH_3_	H	H	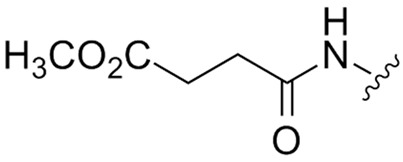	H
**B.**					
**Chemical Name** **(Compound)**	**R**	**R_1_**	**R_2_**	**R_3_**	**R_4_**
4–[(3–carboxypropanoyl)- amino]methylbenzoic acid (**R1**)	H	H	H	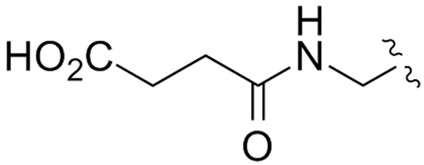	H
4–[(6–carboxycyclohex–3–en –1–yl)carbonyl]amino- benzeneacetic acid (**R2**)	H	H	H	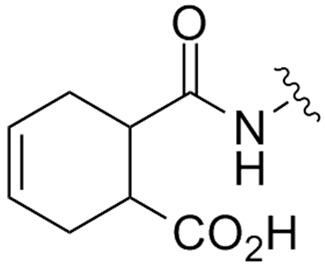	H
5–{[(3–carboxyphenyl)- amino]sulfonyl}–2–methoxybenzoic acid (**R3**)	H	—OCH_3_	H	H	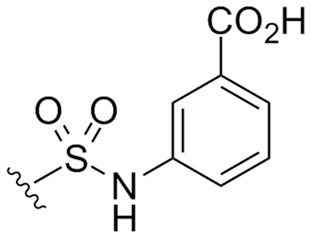
methylene–3,3’–bis–[6–[(1–ethylcarbonyl)amino]-benzoic acid (**R4**)	H	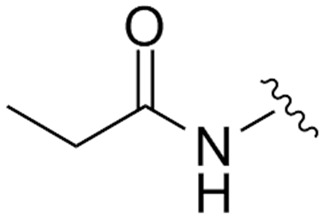	H	H	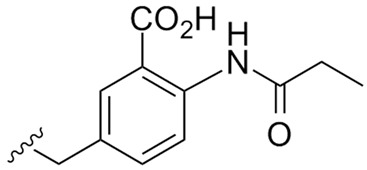
4–[(6–carboxycyclohex–3–en –1–yl)carbonyl]amino-methylbenzoic acid (**R5**)	H	H	H	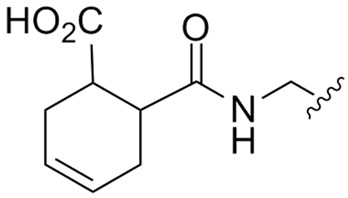	H
4–[1–(4–methoxyphenyl)–1*H*–tetrazol–5–yl]sulfonylmethyl-benzoic acid (**R7**)	H	H	H	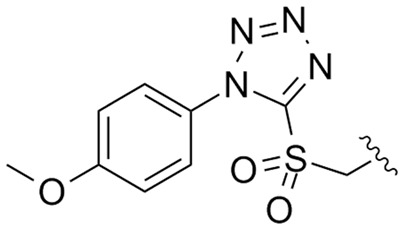	H
4–(1–cyclohexyl–1*H*–tetrazol–5–yl)thiomethylbenzoic acid (**R8**)	H	H	H	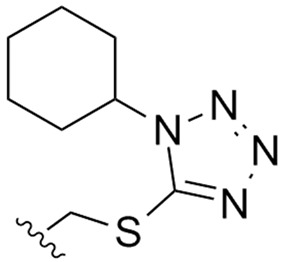	H

**Table 2:** Free energy of binding (Δ*G*_bind_) and binding constant (*K*_i_) estimated for the evaluated compounds.

Molecule	Δ*G*_bind_ [kcal/mol]	*K*_i_ [pM]
**6a**	−13.31	175.3
**6b**	−13.75	83.1
**6c**	−13.73	85.6
**6d**	−12.38	848.7
**6e**	−12.24	1070.0
**6f**	−12.37	862.2
**6g**	−11.90	1890.0
**6h**	−12.87	369.4
**7a**	−14.60	19.8
**7b**	−13.41	148.3
**7c**	−12.80	411.5
**7d**	−13.33	169.4
**7e**	−12.64	539.1
**7f**	−12.36	867.7
**7g**	−12.36	867.5
**7h**	−13.52	123.5

**Table 3:** Selected ADME properties of the new compounds (unit, range of descriptors values for 95% of known drugs). Values out of range are highlighted in red.

Mol	MW	Dipole	SASA	Volume	donorHB	accptHB	Glob	QPlogPo/w	QPPCaco	QPlogBB	#metab	Ro5	Ro3
**6a**	237.2	3.8	481.0	773.7	3	6.5	0.847	0.722	2.2	−2.14	2	0	1
**6b**	237.2	4.2	467.4	762.9	2	5.5	0.864	1.289	3.4	−1.87	3	0	1
**6c**	237.2	4.8	481.3	773.9	3	6.5	0.847	0.723	2.2	−2.14	3	0	1
**6d**	251.2	4.0	499.2	822.4	2	5.5	0.850	1.565	3.4	−1.94	3	0	1
**6e**	291.2	6.3	493.9	808.2	3	6.5	0.850	1.298	2.9	−1.77	2	0	1
**6f**	271.7	4.1	497.0	811.6	3	6.5	0.847	1.185	2.6	−1.92	3	0	1
**6g**	301.7	5.5	535.5	890.7	3	7.25	0.836	1.366	3.0	−1.98	3	0	1
**6h**	363.1	7.9	502.2	825.5	2	5.5	0.847	1.901	3.3	−1.76	2	0	1
**7a**	235.2	5.7	468.3	753.1	3	6.5	0.855	0.696	2.6	−2.03	0	0	1
**7b**	235.2	4.2	453.4	740.1	2	5.5	0.873	1.244	4.0	−1.76	1	0	1
**7c**	235.2	6.9	468.7	753.5	3	6.5	0.854	0.697	2.6	−2.03	1	0	1
**7d**	249.2	3.9	485.8	800.3	2	5.5	0.858	1.519	4.0	−1.83	1	0	1
**7e**	289.2	8.2	481.3	787.3	3	6.5	0.857	1.273	3.5	−1.66	0	0	1
**7f**	269.6	4.9	484.5	791.2	3	6.5	0.854	1.143	2.9	−1.83	1	0	1
**7g**	299.7	6.4	522.4	869.7	3	7.25	0.844	1.325	3.5	−1.88	1	0	1
**7h**	361.1	6.1	487.8	802.9	2	5.5	0.856	1.861	4.0	−1.64	0	0	1

MW—molecular weight (Da, 130–725); dipole—dipole moment (D, 1.0–12.5); SASA—total solvent accessible surface (Å^2^, 300–1000); volume—total solvent accessible volume (Å^3^, 500–2000); donorHB—estimated number of hydrogen bonds donated to solvent (–, 0.0–6.0); accptHB—estimated number of hydrogen bonds accepted by the solute (–, 2.0–20.0); glob—globularity index (–, 0.75–0.95); QPlogPo/w—octanol//water partition coefficient (–, −2.0–6.5); QPPCaco—predicted apparent Caco–2 cell permeability (Caco–2 cells are a model for the gut–blood barrier, predictions are for non–active transport) (nm/s, <25 poor, >500 great); QPlogBB—predicted brain/blood partition coefficient for orally delivered drugs (–, −3.0–1.2); #metab—number of likely metabolic reactions (–, 1–8); Ro5—number of violations of Lipinski’s rule of five (max. 4) [13]; Ro3—number of violations of Jorgensen’s rule of three (–) [15].

## Data Availability

Not applicable.

## Supplementary Materials

The following supporting information can be downloaded at: https://www.mdpi.com/article/10.3390/molecules28052287/s1, Figures S1–S16: Schematic representations of the best computational binding poses for each of the investigated chemical compounds docked to IL–15Rα.
