# Supplementary material for: New, Low–Molecular Weight Chemical Compounds Inhibiting Biological Activity of Interleukin 15
Source: Molecules. 2023 Mar 1;28(5):2287. doi: 10.3390/molecules28052287 (PMC10005041; doi:10.3390/molecules28052287)
Supplement: Supplementary file 1 [file molecules-28-02287-s001.zip › molecules-2205594-supplementary.pdf]

New, Low–Molecular Weight Chemical Compounds Inhibiting Biological Activity of Interleukin 15

Supplementary Materials

Schematic representations of the best computational binding poses for each of the investigated chemical compounds docked to IL–15Rα.

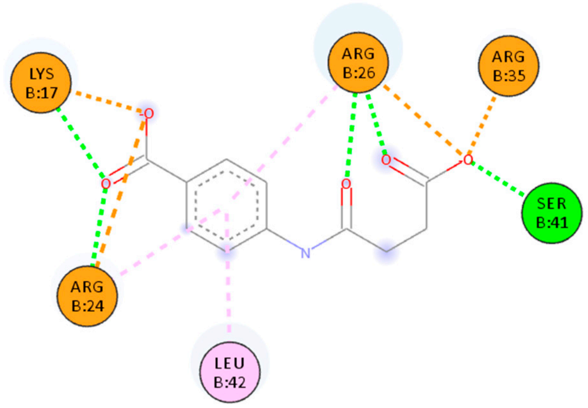

Figure S1. Binding pose of **6a**.

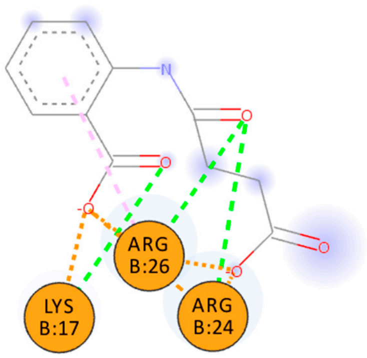

Figure S2. Binding pose of **6b**.

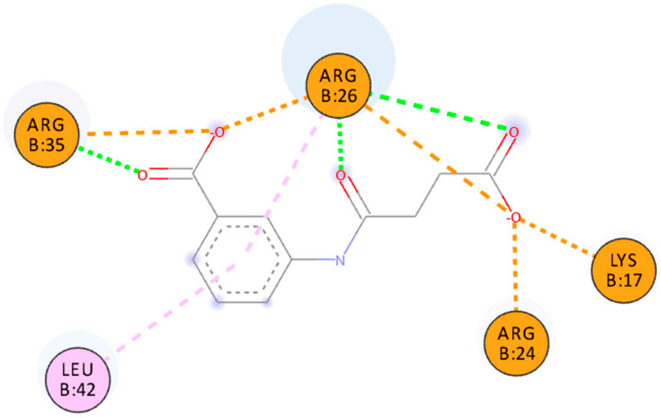

Figure S3. Binding pose of **6c**.

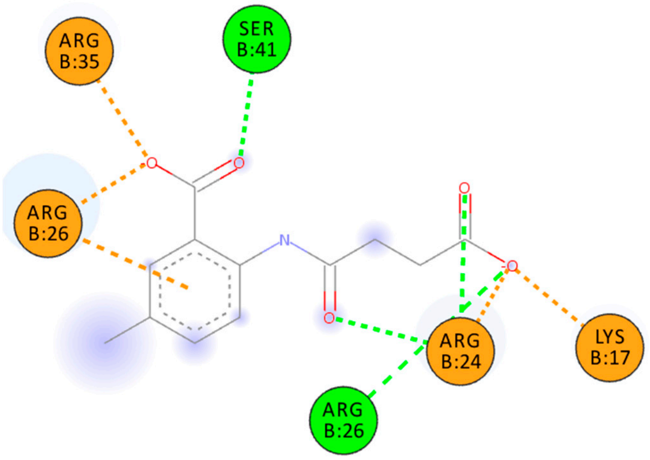

Figure S4. Binding pose of **6d**.

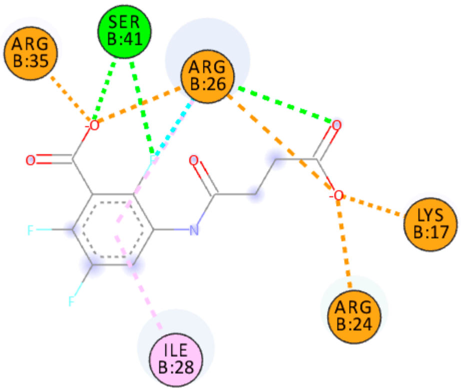

Figure S5. Binding pose of **6e**.

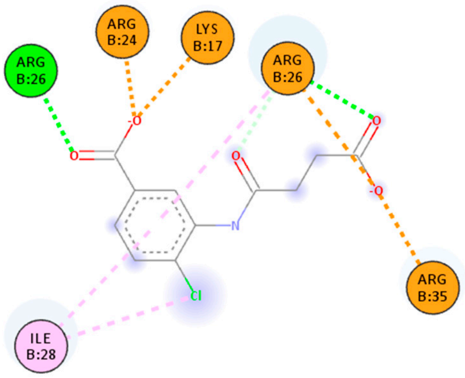

Figure S6. Binding pose of **6f**.

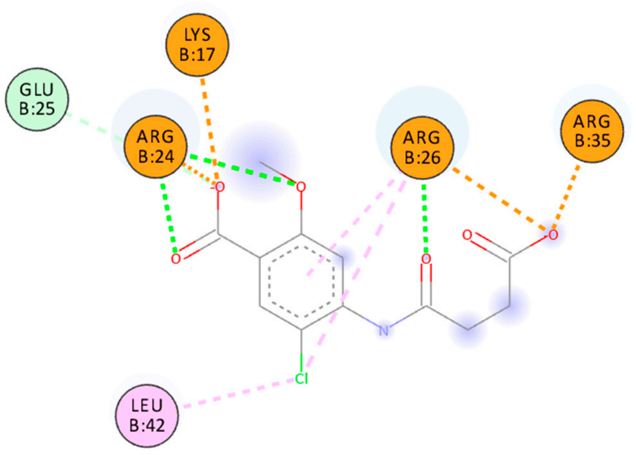

Figure S7. Binding pose of **6g**.

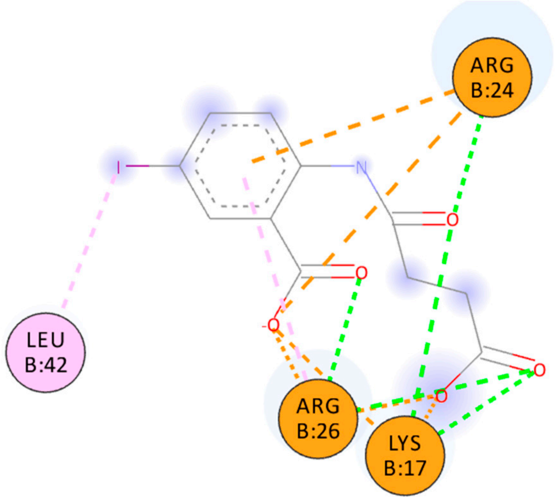

Figure S8. Binding pose of **6h**.

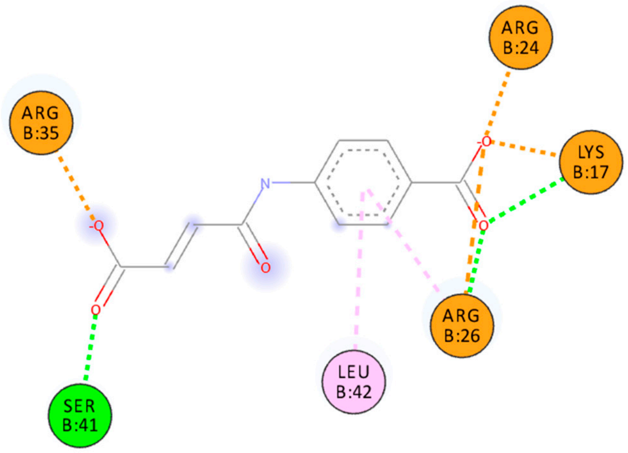

Figure S9. Binding pose of **7a**.

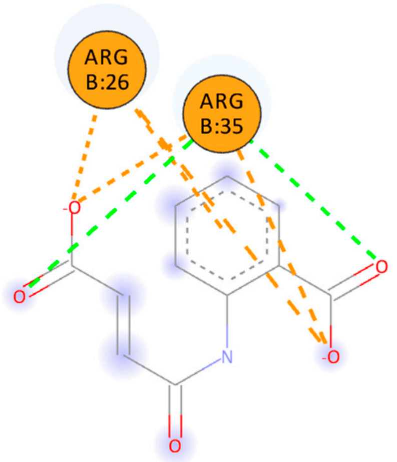

Figure S10. Binding pose of **7b**.

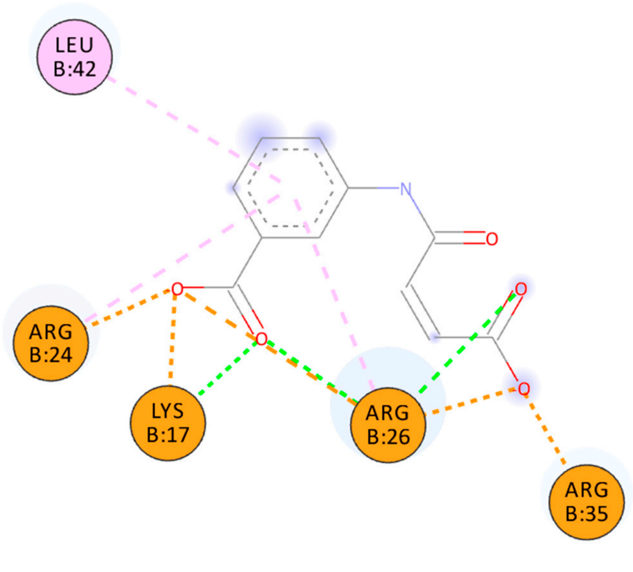

Figure S11. Binding pose of **7c**.

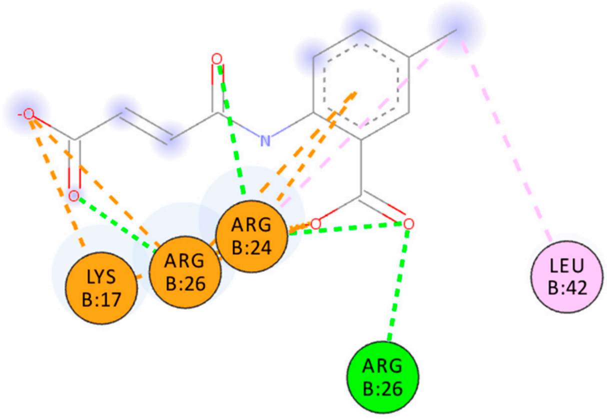

Figure S12. Binding pose of **7d**.

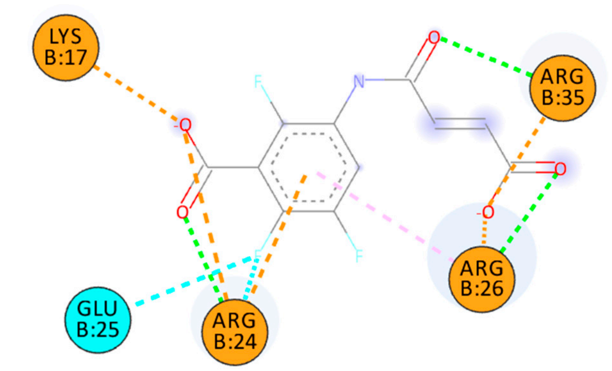

Figure S13. Binding pose of **7e**.

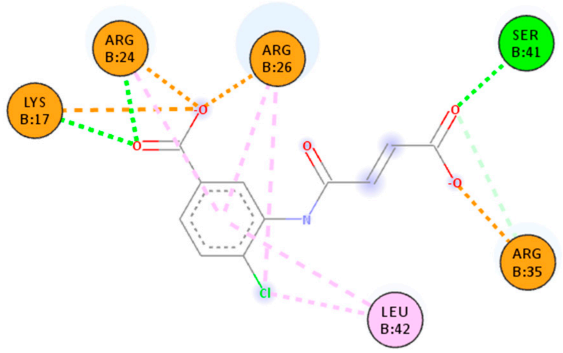

Figure S14. Binding pose of **7f**.

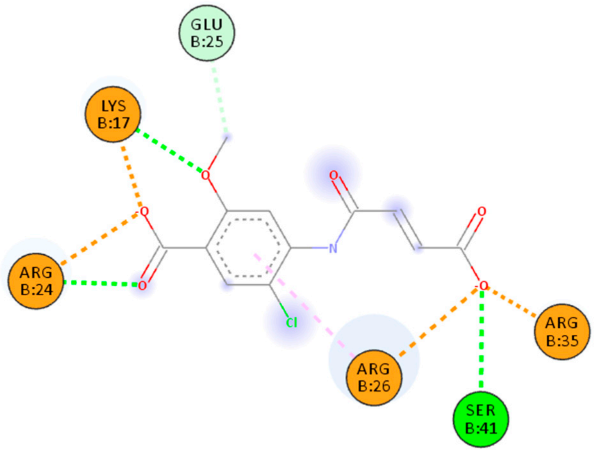

Figure S15. Binding pose of **7g**.

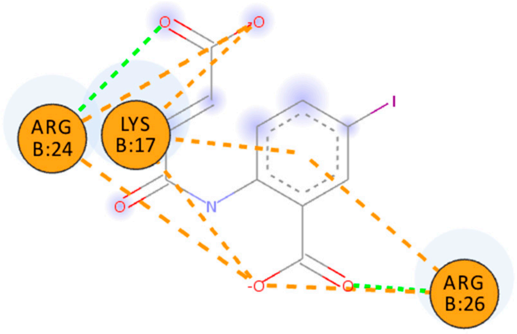

Figure S16. Binding pose of **7h**.
